# Acceptability of identification and management of perinatal anxiety: a qualitative interview study with postnatal women

**DOI:** 10.3389/fpubh.2024.1466150

**Published:** 2024-11-07

**Authors:** Rose Meades, Patricia M. Moran, Una Hutton, Rafiyah Khan, Margaret Maxwell, Helen Cheyne, Amy Delicate, Judy Shakespeare, Kathryn Hollins, Kalpa Pisavadia, Kodchawan (Pim) Doungsong, Rhiannon Tudor Edwards, Andrea Sinesi, Susan Ayers

**Affiliations:** ^1^Centre for Maternal and Child Health Research, School of Health and Psychological Sciences, City, University of London, London, United Kingdom; ^2^King’s Centre for Military Health Research (KCMHR) Institute of Psychiatry, Psychology and Neuroscience (IoPPN), Weston Education Centre, London, United Kingdom; ^3^Nursing, Midwifery and Allied Health Professions Research Unit, University of Stirling, Stirling, United Kingdom; ^4^Institute of Applied Health Research, College of Medical and Dental Sciences, University of Birmingham, Edgbaston, Birmingham, United Kingdom; ^5^Retired General Practitioner, Oxford, United Kingdom; ^6^Surrey and Borders Partnership NHS Trust, Surrey, United Kingdom; ^7^Centre for Health Economics and Medicines Evaluation (CHEME), Bangor University, Bangor, Gwynedd, United Kingdom

**Keywords:** perinatal anxiety, perinatal care, perinatal mental health, acceptability, qualitative research pregnancy, screening, assessment, care pathway

## Abstract

**Background:**

Anxiety in pregnancy and postpartum is highly prevalent but under-recognized and few women receive adequate support or treatment. Identification and management of perinatal anxiety must be acceptable to women in the perinatal period to ensure that women receive appropriate care when needed. We aimed to understand the acceptability to women of how anxiety was identified and managed by healthcare professionals.

**Method:**

We conducted in-depth qualitative interviews with 60 women across England and Scotland approximately 10 months after birth. Women were sampled from an existing systematically recruited cohort of 2,243 women who recorded mental health throughout pregnancy and after birth. All women met criteria for further assessment of their mental health by a healthcare professional. We analyzed the data using a theoretical framework of acceptability of healthcare interventions.

**Results:**

Interview data fitted the seven constructs within the theoretical framework of acceptability. Women valued support before professional treatment but were poorly informed about available services. Services which treated women as individuals, which were accessible and in which there was continuity of healthcare professional were endorsed. Experience of poor maternity services increased anxiety and seeing multiple midwives dissuaded women from engaging in conversations about mental health. Having a trusted relationship with a healthcare professional facilitated conversation about and disclosure of mental health problems.

**Conclusion:**

Women’s experiences would be improved if given the opportunity to form a trusting relationship with a healthcare provider. Interventions offering support before professional treatment may be valued and suitable for some women. Clear information about support services and treatment options available for perinatal mental health problems should be given. Physiological aspects of maternity care impacts women’s mental health and trust in services needs to be restored. Findings can be used to inform clinical guidelines and research on acceptable perinatal care pathways in pregnancy and after birth and future research.

## 1 Introduction

Anxiety is a common mental health issue during and after pregnancy with moderate to severe anxiety affecting an estimated 23% of women during pregnancy and 15% after birth (1). Fewer women meet the criteria for diagnosed anxiety disorders such as generalized anxiety disorder, panic, phobias, or associated conditions like PTSD and OCD (2). Both symptoms and disorders are distressing and debilitating and can significantly impact women, their babies, and their relationships (3, 4). Perinatal anxiety (PNA) can lead to poor fetal development, preterm birth (5), increased risk of further mental health problems for both mother and partner (6), and impaired mother-infant relationship quality (7). It also affects social, cognitive, and emotional development in children (8, 9). Anxiety is often comorbid with depression, and contributes to long-term social and economic costs, with estimates in the UK reaching £8.1 billion for every annual cohort of births (4, 10).

It is crucial to quickly identify and assess problematic PNA so that women can access support or treatment. In this article, treatment refers to psychological therapies and/or medication, while support includes connections with groups or individuals for practical or emotional aid. Successful outcomes rely on several steps: women must enter the healthcare system, their difficulties must be recognized by clinicians, treatment/support must be initiated and adhered to, and symptoms must improve (11). Recent policy and funding have improved perinatal mental health care in the UK (12, 13), leading to changes in service provision (14) and new care pathways (15). However, many women, particularly in underserved groups, still do not access care (16, 17).

Assessing and managing anxiety during and after pregnancy is crucial so healthcare professionals (HCPs) can identify, refer, and treat women with problematic anxiety, leading to better outcomes for both women and infants (8, 18). Research on how acceptable anxiety identification and management are to women is limited, but acceptability is key to success. The World Health Organization considers acceptability essential for any screening program (19), and the UK National Screening Committee lists it as a mandatory criterion for initiating screening (20). Acceptability is also critical in developing high-quality, person-centered care (21), and service-user satisfaction is a key quality indicator in mental health services (22).

Drawing conclusions about the acceptability of perinatal mental health assessment is challenging due to limited research and varying definitions of acceptability, as well as differences in terms, questions, and statements used to measure it (23). Methods for assessing acceptability include uptake rates (24); qualitative approaches, which infer acceptability from terms like comfort, ease, appropriateness, and helpfulness (23); cognitive interviews to explore understanding of assessment questions (25); and self-report surveys measuring usefulness and comfort (26).

The limited qualitative studies on perinatal anxiety (PNA) experiences indicate barriers to treatment include cost, feeling dismissed or receiving inadequate follow-up from healthcare providers (HCPs), focusing on the baby to avoid their own distress, and stigma associated with being seen as a bad mother (27–29). Existing studies feature small samples: six women in Canada (29), 20 in Australia (27), 17 in two English regions (28), and seven in one English region (30). All samples were self-selected, and both English studies occurred pre-COVID-19; experiences and treatment preferences may have evolved since (31).

Research employing a theoretically informed approach to assess the acceptability of perinatal mental health care is scarce but crucial for evaluating healthcare interventions systematically. The Theoretical Framework of Acceptability [TFA; (32)] defines acceptability through seven constructs: affective attitude, burden, ethicality, intervention coherence, opportunity costs, perceived effectiveness, and self-efficacy (see Table 1 for definitions). The TFA can be applied both prospectively and retrospectively by those delivering or receiving interventions. It has evaluated acceptability in perinatal health, including a systematic review of patient-reported measures in maternity care (24), the acceptability of suicidality assessments (25), and exposure therapy for anxiety during pregnancy (33). Employing the TFA can offer insights into women’s experiences with perinatal anxiety assessment and treatment, along with suggestions for improvement.

**Table 1:** Theoretical Framework of Acceptability (TFA) constructs applied to perinatal mental health care pathway.

Acceptability concept	Definition
Affective attitude	How an individual feels about the perinatal mental health care pathway
Burden	The perceived amount of effort that is required to participate in the perinatal mental health care pathway
Ethicality	The extent to which the perinatal mental health care pathway has a good fit with an individual’s value system
Intervention coherence	The extent to which the participant understands the perinatal mental health care pathway and how it works
Opportunity costs	The extent to which benefits, profits or values must be given up to engage in the perinatal mental health care pathway
Perceived effectiveness	The extent to which the perinatal mental health care pathway is perceived as likely to achieve its purpose
Self-efficacy	The participant’s confidence that they can perform the behavior(s) required to participate in the perinatal mental health care pathway

This study aimed to explore women’s acceptability of the care received for perinatal anxiety and their preferences for optimal service provision. While published perinatal mental health care pathways (15) specify five care pathways, we use the term to describe women’s experiences from initial inquiries about mental health to treatment access.

## 2 Materials and methods

A qualitative semi-structured interview study exploring women experiencing perinatal anxiety’s acceptability of being identified and managed in the National Health Service in England and Scotland. We used qualitative methods to understand the experiences of women in more depth than quantitative measures allow and to help identify issues that may not be fully understood through standardized surveys or numerical data.

### 2.1 Study sample

Participants were selected from those that had consented to take part in the longitudinal cohort study Optimizing Care for Perinatal Anxiety. All MAP Alliance participants had previously taken part in an earlier longitudinal cohort study Methods of Assessing Perinatal Anxiety (MAP). The MAP sample is described further here (34) but in brief recruited 2,243 pregnant women in England and Scotland, who completed measures of mental health at ~15 weeks gestation with further data collection at 22 and 31 weeks of pregnancy and 6 weeks after birth. Of these women 794 enrolled in MAP Alliance, a programme of research which explored the health service use of women with and without perinatal anxiety, at 6 months, 1 year and 2 years after birth.

Women were eligible for the current study if they had completed the first MAP Alliance measures at 6 months after birth and had scored positive for anxiety and/or depression at this time point or at any of the data collection points of MAP. We used thresholds on measures recommended by NICE clinical guidelines for assessing perinatal anxiety and depression (the GAD-2 and Whooley questions respectively) (35). Purposive sampling was used in order to establish variation in factors that were likely to influence women’s experiences such as: (i) women from different geographical regions where services may vary; (ii) women from ethnic minority groups; (iii) women whose perinatal anxiety (and/or depression) was/was not identified; and (iv) women who did/did not receive treatment. To ensure all these groups were represented, the sample size was set at 60.

### 2.2 Recruitment

Recruitment took place between August 2022 and March 2023. Eligible women were telephoned by AD or RK to inform them about the study between six and 18 months after birth. If they were interested in the study, they were sent a participant information sheet and consent form. Once a completed consent form was received an interview was arranged by AD or RK. Women who participated received a £20 shopping voucher.

### 2.3 Data collection

Interviews were conducted by female researchers with training and experience of interviewing participants about sensitive topics (AD, RK). Participants knew their interviewer was an academic researcher and were provided with the interview aims. A semi-structured interview schedule was used to guide the interview and explore all aims of the study, but participants were not compelled to answer all questions and likewise, were able to give details of any experience they felt appropriate. Interview topics included disclosure of mental health, assessment, referral, accessing treatment or support, whether women liked or disliked what they experienced, whether their experience fitted with expectations, and whether and how their experience made a difference to them. All women indicating current anxiety or depression were encouraged to talk to their midwife or GP and sent details of support organizations. All interviews were conducted online or by telephone; participants were at work or home. There were no repeat interviews. The average (mean) interview time was 48 min (range 22–85 min). Brief field notes recording pertinent issues for clarification during the interview were not used in analysis. Interviews were recorded on an encrypted device and transferred securely to a third party to be transcribed verbatim. Returned transcripts were anonymised and checked for accuracy by the interviewers but were not returned to participants. A results summary was sent on request.

### 2.4 Data analysis

Transcripts were imported into NVivo12 software (36). Data were analyzed using framework analysis which is suitable for studies where qualitative data is examined within and between different subgroups and to ensure systematic management of a large qualitative data set (37). A combined inductive-deductive coding approach was used which enabled the specific research questions to be addressed and the identification of unexpected or new themes from the data. The framework was developed by PMM and agreed by the research team. Disconfirming data were evaluated throughout analysis.

Anonymised transcripts were coded against the framework by PMM, RM, AD or RK. Any problematic coding issues, additions to the framework, emerging themes and issues requiring resolution were discussed at regular meetings. Reporting follows the guidelines set by the Consolidated Criteria for Reporting Qualitative research [COREQ; (38)].

### 2.5 Ethical approval

Research ethics approval for this study was obtained from the NHS West of Scotland Research Ethics Committee 3 before any recruitment or data collection took place (reference number: 22/WS/0063) Informed consent was obtained from all participants prior to their interview.

## 3 Results

Sample characteristics are presented followed by sections relating to each of the TFA constructs. Direct participant quotes are presented within each (sub)construct. Participant numbers are in the format: Country (E = England, S = Scotland), two-digit recruitment site number (1–17), three digit participant number. Table 2 shows the constructs and themes derived from the theoretical framework of acceptability.

**Table 2:** Constructs and themes related to the seven constructs of the Theoretical framework of acceptability.

1. Affective Attitude
1.1. Availability and accessibility of both maternity and mental health service support
1.2. HCP’s responses to women’s anxiety
1.3. Intervention/service characteristics
2. Burden
2.1. Continuity of care
2.2. Treatment access and engagement
2.3. Service flexibility
3. Ethicality
3.1. Postnatal focus of services on baby and not mother’s needs
3.2. Concerns minimized/not respected
3.3. Acknowledgement of preferences
4. Intervention coherence
4.1. Information about services
4.2. Understanding of the intervention
5. Opportunity costs
5.1. Repercussions/ reputational damage
5.2. Choices taken away/expectations not fulfilled
6. Perceived effectiveness
6.1. Referral process/signposting
6.2. Timeliness of access
6.3. Effectiveness of care/treatments
6.4. Discharge from treatment
7. Self-efficacy
7.1. Confidence in accessing services
7.2. Ability to engage with intervention

### 3.1 Sample characteristics

Initially, 247 women were contacted about the study. Written consent was received from 75 women, of whom 15 did not respond to the interview invitation. The final sample therefore consisted of 60 women (51 in England and 9 in Scotland) who took part in an interview. The sample comprised 12 (20%) women who were Asian, Black, ‘Other’ or unknown, 36 (60%) White British and 12 (20%) White Other. Participants in England were almost equally distributed between geographical regions as defined by National Institute for Health and Care Research (NIHR) Clinical Research Network Areas: North Thames (i.e., North of the Thames but within London), West Midlands, and North East and North Cumbria. Almost all women (58; 97%) were living with a partner, married or in a civil partnership. All women had scored above the NICE recommended threshold for further assessment of: anxiety only (10 women; 16.5%); anxiety and depression (40 women; 67%); depression only (10 women; 16.5%). For 55 (92%) women, their anxiety and/or depression started in pregnancy and for 5 (8%) it arose after birth. Sample characteristics are given in Table 3.

**Table 3:** Sample characteristics.

	*N* (%)
Recruitment Area	
England	51 (85.00)
*London, North Thames*	*15 (25.00)*
*West Midlands*	*18 (30.00)*
*North East and North Cumbria*	*18 (30.00)*
Scotland	9 (15.00)
*Aryshire & Arran*	*1 (1.67)*
*Lanarkshire*	*2 (3.33)*
*Grampian*	*1 (1.67)*
*Greater Glasgow and Clyde*	*2 (3.33)*
*Tayside*	*3 (5.00)*
Ethnic Background	
White British	36 (60.00)
White Other	12 (20.00)
Asian	5 (8.33)
Black	1 (1.67)
Other	1 (1.67)
Unknown	5 (8.33)
Relationship Status	
Living with Partner	18 (30.00)
Married or in Civil Partnership	40 (66.67)
In a Non-Cohabitating Relationship	1 (1.67)
Single	1 (1.67)
Time of First Anxiety/Depression Score > cut-off	
Antenatal	55 (91.67)
*~12 weeks of pregnancy*	42 (70.00)
*~22 weeks of pregnancy*	11 (18.33)
*~31 weeks of pregnancy*	2 (3.33)
Postnatal	5 (8.33)
*~6 weeks postpartum*	4 (6.67)
*~6 months postpartum*	1 (1.67)
Scale Score > cut-off	
GAD-2 (anxiety)	10 (16.67)
Whooley Questions (depression)	10 (16.67)
Both GAD-2 and Whooley Questions	40 (66.67)
Treatment	
Received Health Service Treatment	26 (43.33)^*^
*NHS Psychological Treatment*	*16 (26.67)*
*NHS Medication*	*11 (18.33)*
*Private Psychological Treatment*	*20 (33.33)*
Received No Health Service Treatment	34 (56.67)

* Participants received a combination of listed treatments.

### 3.2 Affective attitude

This construct concerns the ways that women felt about how their anxiety was identified and managed. Feelings are reported about access to and availability of services, HCP responses to women’s anxiety and about services offered.

#### 3.2.1 Availability and accessibility of maternity care and mental health service support

This theme reflects affective attitudes to both the perinatal mental health pathway and the maternity care women received because this care had the potential to positively or negatively impact anxiety. Feelings were positive about services that were perceived as providing women with appropriate ease of access, even if that service was not eventually needed:

*I had all the (psychiatric support) numbers I needed, I knew what I needed to do […] Knowing that the help is there if I need it. That was helpful. I needed to know that I can get that if I need to. (E04232).*

Services that provided care tailored to individuals’ needs were also considered favorably. This included maternity care. For example, after previous pregnancy loss, there was potential to reduce anxiety through additional scans:

*Because of the early bleeding I had an extra three scans I think, or four maybe. […] That definitely helped. The weeks following the scans I was definitely in a better mood and wasn’t as stressed. (S01022).*

However, if maternity care was perceived as poor or lacking, anxiety could increase:

*I lost some blood, but because of when it happened, because of Covid, it was really difficult to get a scan and it just really scared me. I think I was completely sure I was having another miscarriage. From that point until about 13 or 14 weeks in the pregnancy I just felt really, really anxious. (E01009).*

Women also felt that their anxiety could worsen if told that a service would be provided but it was not, for example providing a telephone number but not answering the phone:

*When you are that anxious, you want that touch point on speed dial, almost, that professional where you can go, oh is this normal? Is this okay? Am I okay? […] but then when she (midwife) did not reply it was worse. […] You’d try and call the service, and then they would not pick up…that would actually add to the anxiety, rather than alleviate it. (E01081).*

#### 3.2.2 Healthcare professionals’ responses to women’s anxiety

The way in which HCPs responded to women who disclosed their anxiety impacted women’s feelings about care received. For example, implying that anxiety may be damaging to the baby could unintentionally add further anxiety:

*I had to then talk to a consultant or a doctor, on the phone. And it was just one thing they said to me, and I’m sure they did not mean it like they said it, but it was almost, they said because I’m in such a high state of anxiety, that I could be hurting the baby. That really, really upset me […] it just made it (anxiety) a hundred times worse. (E05033).*

Similarly, telling women that they should enjoy their pregnancy was unhelpful:

*He (CBT therapist) kept saying, … how we needed to be enjoying this time as a couple because it was never, ever going to be the same again as soon as the baby was born […] I was just like, this is making me feel worse, more anxious that I’m not making the best of the time now. (E12127).*

Acknowledgement of anxiety and calm words of reassurance were helpful and made women feel positive about the care they were receiving, whether a health visitor who was a *‘motherly figure’* affirming that *‘everything [I] was doing was okay’ (E01077)* or a psychiatric nurse providing more intensive support:

*She (psychiatric nurse) used to come and see me every week. She made me feel normal, she made it sound like, “It’s no big deal, everything’s fine, this is a normal way for you to feel, given what you have been through.” And just a bit of sympathy and empathy about what had happened before. (E05002).*

#### 3.2.3 Health service characteristics

Women were concerned about the structure of health services, for instance there was a sense of t concern and anxiety about lack of continuity of midwife because this involved repeating their story multiple times to different people that they had no relationship with:

*From 24 weeks on, every single appointment I said, this is the first time I’m meeting you (midwife), this makes me feel really anxious, it makes me feel upset. (E01035).*

Conversely, when women experienced continuity it provided safety and comfort which facilitated honest conversations about mental health:

*It wasn’t a tick-list, where they go, how is your mood? It was more, she (midwife) was sitting in front of me, and she looked at me and she went, are you okay? Because she’d seen me at all those previous appointments, she knew that there was a change in the way I was […] I’m glad that it was always her. (E04352).*

There was also an awareness that perinatal mental healthcare would end at a specified timepoint in some services, which caused worry about options for future support or the possibility of having to cope alone:

*I feel like there is a degree of anxiety at the moment, as soon as the baby turns one, then I do not have the Perinatal Service anymore. So, I then feel like I’m alone with it all again. (E12127).*

### 3.3 Burden

Burden concerns the perceived amount of effort that was required by an individual to engage with the perinatal mental health care pathway and can be seen as a measure of effort needed to overcome barriers to care. Women spoke about the amount of effort it took to deal with multiple different HCPs and to access services.

#### 3.3.1 Continuity of carer

Women were dismayed by having to tell their story or circumstances to multiple HCPs. Sometimes this effort dissuaded women from engaging with services at all:

*I just feel like again I’m going to see four or five different people that all do not know me. I’ll have to re-explain the same stuff, over and over again. I’m never going to get anywhere, so I might as well just keep trying to deal with it myself. (E04098).*

Ongoing care from the same HCP encouraged engagement and disclosure of feelings:

*I had the same midwife every time…so she got to know me, and I had that kind of comfortable relationship with her where I felt like I could say, “Oh, actually, I’ve been a bit anxious this week,” or, she was able to pick up on things, that someone who had not seen me before might not have. (E12113).*

#### 3.3.2 Treatment access and engagement

If women were told to self-refer to or call a service themselves, the experience could be inconvenient because it was hard to find time whilst caring for a new baby, potentially other children, and being sleep-deprived. Self-referral could be difficult because of not knowing what to say or how to navigate entry into a service, or considered to be pointless because the long waiting list could mean that a pregnant woman’s baby would be born before she could access therapy:

*They say that they have a mental health support team they could have referred me but the wait was about six months. Given that I started talking with the midwife at three months, in the pregnancy, it (referral) was entirely irrelevant. (E01009).*

If services could offer some flexibility in offering online or in-person appointments, or within the times that they could visit women at home, this reduced the burden of engagement:

*She’d (HCP) always made it around the time that I wanted, and that was really good. […] I think she did set days, but yeah, the times were quite flexible. (E05033).*

### 3.4 Ethicality

This construct reflects the extent to which the perinatal mental health care pathway fitted with women’s value systems, or what was considered important to women in perinatal mental health care. Overarchingly, women considered that there should be as much focus on mothers as individuals as on babies, that HCPs should listen to women and treat them as individuals and should listen and respect preferences for care and treatment.

#### 3.4.1 Focus of postnatal services on baby not mother

Women felt that postnatal care/service focused only on the baby and did not account for their own needs. For some women, support that incorporated their baby worked well but for others individual care was needed:

*…There wasn’t any service where I as a person could be dealt with. It felt like the only options were related to your kid, and you have to go to a Children’s Centre and sit on a carpet with your kid, and they’d talk to you about that. (E01025).*

#### 3.4.2 Concerns minimized or not respected

Women valued being treated with respect as an individual who should be listened to. When women were asked questions about their wellbeing or mental health, they wanted this to be done with meaning, not simply as a requirement:

*Anything I said additionally around like how I felt, it wasn’t that it was ignored, but it was, I mean every midwife appointment I had they asked the question, like how do you feel, but it was more the let us tick that box on the form and make sure I’ve asked that question. (E06064).*

#### 3.4.3 Acknowledgement of preferences

Women wanted their own values and beliefs to be acknowledged by HCPs. Decisions about parenting and medication for example were sometimes not respected such as a GP telling a mother to let her baby cry when she did not want to and was very distressed by this, and a psychiatrist who did not acknowledge a woman’s wishes not to be treated with medication immediately:

*I used to dread going (to the psychiatrist), I used to feel like I wasn’t listened to. She was always pushing medication, which I repeatedly said that at first I wanted to try without. (E02002).*

Women valued scenarios in which they felt listened to, and this encouraged them to engage with services and attend appointments:

*I was offered a referral to the perinatal mental health team, I kind of voiced that I wasn’t sure, wasn’t really that confident with being put on medication. I was assured that’s not the only thing that they would be able to support us with. So, they seemed to understand. (S01024).*

### 3.5 Intervention coherence

This theme relates to women’s understanding of the care pathway and of treatment received. Women described how important it was to know about what services were available and what would happen along the pathway, yet this information was often missing.

#### 3.5.1 Information about services

Most women recognized that they would benefit from some level of support, but they were unable to specify what that help could be. It was therefore important for HCPs to clearly articulate the pathway that women would access.

Participants also expressed some confusion around specific services that were available to them as well as the estimated waiting time for help and support. For example, women did not know whether they would be referred to specialist perinatal metal health or generic Talking Therapy services (psychological treatments that involve discussing mental health concerns with a trained therapist including cognitive behavioral therapy and counseling). This was particularly confusing for women whose first language was not English:

*The information she (midwife) gave me was not, was insufficient. I’m not sure whether it’s useful for me or not. […] They just provide me the numbers, and saying that you should seek help if you think you need it. But I’m not sure … they would just say it’s a psychiatric service, but I’m not sure what is going to happen. […] we are new to the country. I had no idea what I … and under what circumstances I should seek GP’s help, I do not know. Honestly, I do not know, I do not even know now. (E01042).*

In order to make an informed decision when seeking help, women required clarity on waiting times and availability of different types of support. When HCPs took time to explain a pathway, the impact of understanding the process was reassuring:

*We spoke about things like medication and talking therapy and what support they can offer and how long they’ll be there and how they can support me after baby arrives and all this kind of stuff and just talked about the whole journey so that I sort of knew what to expect, that was wonderful. (S01024).*

#### 3.5.2 Understanding interventions

Once enrolled in treatment, women did not necessarily need to understand what the treatment was to engage in it successfully:

*I’m not sure, really, what things come under CBT because it was all integrated in our sessions, it wasn’t like, “Today, we are going to do CBT.” She’d come round and we’d talk. (E05002).*

Understanding that they would be given time, would not be judged, would be listened to and could talk openly and honestly to someone who understood what they were experiencing were important to women:

*I could say things to her that might sound completely ridiculous to someone else but I knew she would take me seriously about it as well and understand how I was feeling. (E10090).*

### 3.6 Opportunity costs

This construct concerns the perceived costs or detriment to an individual of participation in the perinatal mental health care pathway. Costs were largely related the ‘bad mother’ stigma which was preeminent amongst women who debated speaking with a HCP because *“they might think that I cannot look after my baby or something” (E05084).* Some women endured their own costs of paying for private treatment outside of the NHS as they did not feel that the NHS was meeting their needs.

#### 3.6.1 Repercussions/reputational damage

In addition to feeling judged by HCPs and by family or friends, women also feared not being able to return to their career or experiencing reputational damage should they disclose how they were feeling to HCPs:

*I do not feel able, I did not feel able to reach out to the GP and say that I feared I had postnatal depression, because I then would not be able to go back into my job, potentially. (E04332).*

#### 3.6.2 Choices taken away/expectations not fulfilled

Further costs related to the available treatments being limited in scope and not being able to access the type of treatment that women felt was required. One woman who reached out for support felt that the offer of CBT was not appropriate for her, but nothing else was available:

*The IAPT Team and the triage and everything, just decided that it was CBT that I was having and I kind of felt at the time, that I was hoping that I would get more of a counselling- based service, but yes, I did not get a choice, they just said that’s what I was having… I do not really feel like that helped me…I was kind of, felt like I was a bit stuck with what I got. (E12127).*

### 3.7 Perceived effectiveness

Women’s views about how effective the pathway was for them in supporting their mental health related to the referral process, waiting times, treatment and medication, and the end of care. Perceived effectiveness was impacted by previous experience and participants who had poor experiences in NHS settings were less likely to disclose poor mental health and access additional support. This was especially the case for women who had previous traumatic experiences including loss and difficult birth experience. Such experiences undermined the trust they had in health care providers with some explicitly describing feeling unsafe.

#### 3.7.1 Referral process and signposting

As women often did not know what services or support may be available, they relied on HCPs to help them navigate this. This did not have to be clinicians but could also be ancillary staff:

*She (GP receptionist) just literally asked me, what’s the call about? I explained what I wanted to talk about. She was lovely, she was really professional, she was like, “Actually, I think there’s a service that you would be better suited to,” explained what it was, and she was really nice and I was like, no, that does sound better…she was perfect. (E06108).*

#### 3.7.2 Timeliness of access

Expectations were of long waiting times to access care, which impacted women in different ways. Some turned to private healthcare options which were also perceived as providing choice and autonomy over the type of treatment preferred. Those with pre-existing symptoms of anxiety who were already accessing private mental health support continued through their pregnancy and benefited from the existing trusting relationship with a therapist. For those who did try to access NHS care, some women experienced a long wait, and others were surprised when they were able to see a specialist within several weeks:

*I did a self-referral and then had a triage call, a week later. So, it was pretty quick this time and then I, I think I started therapy three weeks after that, four weeks after that. So, it was very efficient. I know if you are on the perinatal pathway, you are seen sooner, but I was surprised how soon I was seen. (E04277).*

#### 3.7.3 Effectiveness of care

When discussing how anxiety was managed, women cited the importance of emotional and practical support from their partners, family, friends and existing networks foremost. Existing networks also enabled attendance at appointments for further support. Attending groups and talking to peers was particularly useful for participants as it allowed them the opportunity to discuss anxieties and normalize their thoughts. In some cases, HCPs had facilitated support groups by signposting women to local groups, NCT courses and setting up WhatsApp groups. Women who felt that they needed something more than a support group sometimes found that being able to talk with a HCP frequently was effective:

*I reached out to her (Health Visitor) and said, you know, “I keep having really bad thoughts.” And she was amazing, she was like, “I’ll do weekly calls with you, or I can come round to your house every week, and we can just talk about it.” She did offer to refer me for Talking Therapy but, actually, just having her there really helped. (E1077).*

Those who utilized talking therapies discussed the benefits of engaging in therapy and understanding their own mental health. They also highlighted points for improvement which included the need for individual sessions rather than group, flexibility to participate remotely or face to face, and reducing the use of therapy ‘homework’ exercises. Participants expressed a preference for mental health support by professionals with expertise in perinatal issues rather than general mental health support as this was thought to be more beneficial. Some felt that the generic talking therapy they were offered was ineffective, with cited reasons being that it focused more on daily functioning, which was not the key problem, or because therapy was time-limited or its effects did not last:

I think a lot of the [CBT] stuff never had a long-lasting impact. […] and ultimately I think it’s about trying to deal with the root cause. (E01081).

#### 3.7.4 Discharge from treatment

A further aspect of perceived ineffective treatment was being discharged from care too soon. Women feared being left to cope with problems alone, and questioned why services could not be flexible in providing less frequent appointments but over a longer period:

*I’d got to a point where, because I was phoning her, I was on the phone to her weekly, a lot had not really changed in my life. So, I probably would have preferred her, rather than discharging me, turn it to more infrequent calls. So, you know, just popping in, “I’m just checking in, how are you doing?” (E07192).*

### 3.8 Self-efficacy

In this context, self-efficacy concerns women’s confidence in being able to engage with or perform the necessary behaviors to take part in the perinatal mental health care pathway.

#### 3.8.1 Confidence in accessing services

If women overcame concerns about implications of engaging with services, a period followed of building up to making a telephone call or speaking with a HCP. This was facilitated by having an ongoing or trusting relationship with an HCP:

*I think at that point I knew I could call up, I called up the talking therapies team and they signed me up to a new mothers and baby group which was really, really good. (E10090).*

However, not all women were confident in speaking with services; for example, women whose first language was not English or who had recently come to the UK found navigating services difficult:

*I did talk to the midwives, they offered me, like I say, if you want to you can do a clinical service, attend a psychiatric service. And, then they provide me the information, but it’s … But it seems like the information they give me is overwhelming. Well, like you have to apply (to) the clinical service yourself. … I mean the procedure is quite difficult for me so I do not want to do it. So it ended up I did not seek. (E01042).*

#### 3.8.2 Ability to engage with intervention

Once women had entered a service, confidence in engaging was high, although there were specific processes that were difficult such as completing CBT ‘homework’ due to lack of time or energy, and technological difficulties with online appointments:

*It was very, “Oh, you need to do the homework and you need to do the work, otherwise it will not work,” and it was like, well, I’m really struggling… because I cannot even manage to get ready, get dressed some days. […] It was just making things worse really and I think it would have been better if he had approached it, as like, “What do you feel you could do, as the first step?” Then it might have just been something really tiny, that I felt like I could build it from there. (E12127).*

## 4 Discussion

This study assessed the acceptability of perinatal mental health care in 60 women recruited early in pregnancy, whose babies were at least 6 months old. It is the largest qualitative study exploring the views of women from three regions in England and Scotland, all of whom exceeded the threshold on NICE-recommended anxiety or depression measures during the perinatal period. It is also the first to examine the care pathway from initial disclosure to treatment, using the seven constructs of the TFA.

Key findings include: (i) in the domain of affective attitude, maternity care experiences influenced women’s feelings about mental health services; (ii) in intervention coherence, women had little awareness of available mental health support; (iii) in opportunity costs, stigma around being perceived as a bad mother or fears of child removal discouraged care-seeking; and (iv) in ethicality, women often preferred support and individualized care over formal ‘treatment.’ These aspects will be discussed further.

### 4.1 Impact of maternity care on perinatal mental health

Evidence links HCP support during birth with birth trauma development (39), and this research adds that HCP actions and service function throughout pregnancy also affect anxiety. Negative impacts included women not feeling safe after previous negative maternity experiences and distress from communication issues with HCPs. Shortfalls in maternity care raised anxiety levels, deterring women from accessing care. Thus, assessing maternity care quality and its association with mental health and engagement is crucial for system-level improvements, yet suitable patient-reported experience and outcome measures remain limited (40). This study also highlighted the importance of trusted relationships with HCPs, which facilitated mental health conversations. This aligns with research showing that continuity of carer enhances trust, improves satisfaction, boosts engagement, and may reduce perinatal anxiety and depression (41–43).

### 4.2 Information giving and concern about impact of help seeking

Mental health literacy plays a key role in seeking help for perinatal mental health (44). Women in this study recognized their symptoms and the need for support, which aids help-seeking. However, they found local information about support and treatment services insufficient and were unsure about the next steps if they sought help. It’s crucial that women are aware of available services. Providing appropriate information from HCPs would benefit women by: (i) helping them make informed choices about mental health care, improving engagement (41); (ii) enabling women with milder symptoms to self-refer and access support faster; and (iii) improving understanding of the care pathway, thus enhancing acceptability and engagement. Understanding the pathway is vital, as many women in this study worried that disclosing mental health issues might be seen as a risk to their unborn child. Anxiety may invite stigma like invalidation and minimizing (45), a common finding in perinatal mental health research. Disclosure of distress often triggers guilt, shame, fears of being judged by HCPs, and concerns about social services involvement and child custody loss (46, 47). Despite increased funding and awareness in perinatal mental health, women remain afraid to seek help. Reducing stigma and ensuring HCPs are approachable, empathic, and non-judgmental is essential for women to feel comfortable disclosing distressing feelings.

### 4.3 Support before treatment

Women in this study emphasized the need for support before or alongside treatment. Previous research also indicates that perinatal women value initial support, including from existing networks, before seeking professional intervention (48). However, public opinion differs, with research showing that professional interventions like counseling and psychotherapy are viewed as more helpful, with partner/family support seen as less valuable (44). This narrative is important because it may undermine social support as a valid intervention for mild to moderate perinatal anxiety. Research often focuses on individual women, placing responsibility on mothers rather than addressing the need for family and system-level interventions that target social determinants of mental health (49).

Additionally, this view may delay recovery by channeling women into services with waiting periods when other support options could be available sooner. While specialist treatment is often needed for perinatal mental health issues, evidence suggests that peer support and non-specialist interventions by HCPs, such as midwives, can effectively prevent or treat perinatal anxiety (50). These non-specialist interventions align with the World Health Organization’s call for “evidence-based, cost-effective, and human rights-oriented mental health and social care services in community-based settings for early identification and management of maternal disorders” (51).

### 4.4 Strengths and limitations

This is the first study to report findings from postnatal women across England and Scotland recruited early in pregnancy, all meeting NICE criteria for further mental health assessment. The TFA offered a structured, theoretically informed approach to assess acceptability of perinatal mental health care. A limitation is the relatively well-educated sample, likely contributing to higher mental health literacy. Although there was some ethnic diversity, future research should examine more ethnically and socio-economically diverse groups. The TFA framework addressed all seven constructs of acceptability, but the inclusion of a safety and risk construct could be beneficial, as concerns about unsafe care were not fully captured, consistent with findings in surgery (52).

## 5 Conclusion

Acceptability of healthcare influences access to, engagement with, and completion of healthcare pathways. This study highlights that in a national sample of postnatal women meeting criteria for further mental health assessment, the perinatal healthcare pathway needs optimization. Key improvements include fostering trust with healthcare providers to encourage mental health discussions, providing information to reduce stigma and fears of child removal, endorsement and funding of perinatal support services as well as specialist perinatal mental health care and improving and measuring maternity care quality.

## Data Availability

The datasets presented in this article are not readily available because of confidentiality requirements. Requests to access the datasets should be directed to the corresponding author.
