# Supplementary material for: Acceptability of identification and management of perinatal anxiety: a qualitative interview study with postnatal women
Source: Front Public Health. 2024 Nov 7;12:1466150. doi: 10.3389/fpubh.2024.1466150 (PMC11579707; doi:10.3389/fpubh.2024.1466150)
Supplement: Supplementary file 1 [file Table_1.DOCX]

**Acceptability of identification and management of perinatal anxiety:**

**A qualitative interview study with postnatal women**

Completed consolidated criteria for reporting qualitative studies (COREQ) 32-item checklist

| **No** | **Item** | **Manuscript location** | **Guide questions/description answered in the manuscript** |  |
| --- | --- | --- | --- | --- |
| **Domain 1: Research team and reflexivity** |  |  |  |  |
| Personal Characteristics |  |  |  |  |
| 1. | Interviewer/facilitator | Methods, section 2.3 data collection | Which author/s conducted the interview or focus group? |  |
| 2. | Credentials | Title page | What were the researcher's credentials? *E.g. PhD, MD* |  |
| 3. | Occupation | Methods, section 2.3 data collection | What was their occupation at the time of the study? |  |
| 4. | Gender | Methods, section 2.3 data collection | Was the researcher male or female? |  |
| 5. | Experience and training | Methods, section 2.3 data collection | What experience or training did the researcher have? |  |
| Relationship with participants |  |  |  |  |
| 6. | Relationship established | Methods, section 2.3 data collection | Was a relationship established prior to study commencement? |  |
| 7. | Participant knowledge of the interviewer | Methods, section 2.3 data collection | What did the participants know about the researcher? e*.g. personal goals, reasons for doing the research* |  |
| 8. | Interviewer characteristics | Methods, section 2.3 data collection | What characteristics were reported about the interviewer/facilitator? e.g. *Bias, assumptions, reasons and interests in the research topic* |  |
| **Domain 2: study design** |  |  |  |  |
| Theoretical framework |  |  |  |  |
| 9. | Methodological orientation and Theory | Methods, section 2.4 Data analysis | What methodological orientation was stated to underpin the study? *e.g. grounded theory, discourse analysis, ethnography, phenomenology, content analysis* |  |
| Participant selection |  |  |  |  |
| 10. | Sampling | Methods, section 2.1. Study sample | How were participants selected? *e.g. purposive, convenience, consecutive, snowball* |  |
| 11. | Method of approach | Methods, section 2.2. Recruitment | How were participants approached? e*.g. face-to-face, telephone, mail, email* |  |
| 12. | Sample size | Results, section 3.1 Sample characteristics | How many participants were in the study? |  |
| 13. | Non-participation | Results, section 3.1 Sample characteristics | How many people refused to participate or dropped out? Reasons? |  |
| Setting |  |  |  |  |
| 14. | Setting of data collection | Methods, section 2.3 Data collection | Where was the data collected? e*.g. home, clinic, workplace* |  |
| 15. | Presence of non-participants | Methods, section 2.3 Data collection | Was anyone else present besides the participants and researchers? |  |
| 16. | Description of sample | Results, section 3.1 Sample characteristics. Table 2. Sample characteristics | What are the important characteristics of the sample? *e.g. demographic data, date* |  |
| Data collection |  |  |  |  |
| 17. | Interview guide | Methods, section 2.3 Data collection. | Were questions, prompts, guides provided by the authors? Was it pilot tested? |  |
| 18. | Repeat interviews | Methods, section 2.3. Data collection | Were repeat interviews carried out? If yes, how many? |  |
| 19. | Audio/visual recording | Methods, section 2.3. Data collection | Did the research use audio or visual recording to collect the data? |  |
| 20. | Field notes | Methods, section 2.3. Data collection | Were field notes made during and/or after the interview or focus group? |  |
| 21. | Duration | Methods, section 2.3. Data collection | What was the duration of the interviews or focus group? |  |
| 22. | Data saturation | No | Was data saturation discussed? |  |
| 23. | Transcripts returned | Methods, section 2.3 Data collection | Were transcripts returned to participants for comment and/or correction? |  |
| **Domain 3: analysis and findings** |  |  |  |  |
| Data analysis |  |  |  |  |
| 24. | Number of data coders | Methods, section 2.4 Data analysis | How many data coders coded the data? |  |
| 25. | Description of the coding tree | Methods, section 2.4 Data analysis and Table 3. Constructs and themes related to the seven constructs of the Theoretical framework of acceptability | Did authors provide a description of the coding tree? |  |
| 26. | Derivation of themes | Methods, section 2.4 Data analysis | Were themes identified in advance or derived from the data? |  |
| 27. | Software | Methods, section 2.4 Data analysis | What software, if applicable, was used to manage the data? |  |
| 28. | Participant checking | Methods, section 2.3 Data collection | Did participants provide feedback on the findings? |  |
| Reporting |  |  |  |  |
| 29. | Quotations presented | Section 3. Findings. All sub-themes have supporting quotes | Were participant quotations presented to illustrate the themes / findings? Was each quotation identified? e*.g. participant number* |  |
| 30. | Data and findings consistent | Data (quotes) represent each subtheme and themes throughout Section 3. Findings | Was there consistency between the data presented and the findings? |  |
| 31. | Clarity of major themes | Results, section 3 reports constructs of acceptability and themes under their own named subheadings | Were major themes clearly presented in the findings? |  |
| 32. | Clarity of minor themes | Methods, section 2.4 Data analysis states that disconfirming data was evaluated throughout. | Is there a description of diverse cases or discussion of minor themes? |  |
